# Challenges to Improve the Stability and Efficacy of an Intravesical BCG Product 

**Published:** 2014

**Authors:** Hamidreza Hozouri, Dariush Norouzian, Nastaran Nafissi-Varcheh, Reza Aboofazeli

**Affiliations:** a *Department of Pharmaceutics, School of Pharmacy, Shahid Beheshti University of Medical Sciences, Tehran, Iran.*; b *Department of Nanobiotechnology, Pasteur Institute of Iran, Tehran, Iran.*; c *Department of Pharmaceutical Biotechnology, School of Pharmacy, Shahid Beheshti University of Medical Sciences, Tehran, Iran. *

**Keywords:** Bacillus Calmette-Guérin, BCG, Intravesical, Lyophilization, Trehalose, *Mycobacterium bovis*

## Abstract

The aim of this investigation was to improve the storage stability and survival rate of an intravesical BCG product, manufactured with an attenuated strain of *Mycobacterium bovis *(Pasteur strain 1173P2 of BCG) in the presence of sodium glutamate. Formulations with various concentrations of trehalose (a known protectant) were developed as liquid and lyophilized forms. Formulations were evaluated by different methods, including optical density measurement, safety assessment, skin reaction test, moisture content determination, viability assay, bacterial and fungal contaminations and the results were compared with those obtained for sodium glutamate-containing formulations. The stability tests were also carried out in various storage durations and different temperatures. To develop the lyophilization protocol, glass transition temperatures in the presence of both stabilizers were determined using differential scanning calorimetry. In general, results showed that trehalose could considerably increase the stability of the product against freezing and drying processes, increase the survival rate even in the liquid formulations, as well as the production of an acceptable cake. However, further studies are required to optimize the product characteristics.

## Introduction

It is more than a century that bacteria have been used as a potential therapy for cancers, due to the tumor regression observed in individuals with concomitant bacterial infection. In the early 1890s, Coley (an American surgeon) observed that in certain cancer patients, the tumor would regress following accidental bacterial infections. Based upon this observation, he used live bacteria and their toxins for the treatment of cancers, and finally developed a safe bacterial production in order to simulate an infection with the accompanying fever and successfully treat various types of cancers. In 1929, Pearl (at Johns Hopkins Hospital) noticed that patients who suffered from tuberculosis showed significantly less malignant tumors than a control group (1-4). 

Non-pathogenic bacterial species (live attenuated or genetically modified) are now being explored as potential antitumor agents, and are capable of providing direct tumoricidal effects or delivering tumoricidal molecules, leading to the inhibition of tumor growth. 

Bacillus Calmette-Guérin (BCG), a live attenuated form of *Mycobacterium bovis*, was first used intravesically for the cancer therapy in 1930s and has been considered to be the most effective agent against carcinoma *in situ *(CIS) of bladder or superficial bladder tumors. BCG prevents or reduces tumor recurrences, tumor progression, improves survival over surgery alone and decreases the need for subsequent cystectomy (5-11). 

Domestically produced Intravesical (Immune) BCG is an injectable suspension which contains sodium L-glutamate monohydrate (1.5% w/v) and an attenuated strain of *Mycobacterium bovis *(Pasteur strain 1173P2 of BCG) in which the number of culturable particles is between 32-128 × 107/mL (96-384 × 107/dose). This product should be stored in freezer at the temperature of less than -10 °C and its mean shelf life at this condition is 12 months, whereas other commercially available BCG intravesical products which are prepared with various strains of *Mycobacterium bovis *possess longer shelf-lives while stored at refrigerator temperature. On the other hand, the frozen product should be thawed before administration, resulting in a considerable increase of the dead mass and in turn a decrease in its efficacy. 

The aim of this research was to improve the formulation of BCG intravesical in order to provide a longer stability (i.e. shelf-life) and a better efficacy. We hypothesized that changing the stabilizer and using lyophilization technique could have probably considerable impacts on both the stability and efficacy of this product. Therefore, trehalose, a non-reducing disaccharide that is abundant in mycobacterial cell wall (12), was selected as an organism protectant for the preparation of BCG intravesical product. The efficacy and stability of these new formulations, in both liquid and freeze-dried forms, were evaluated and compared with both liquid and lyophilized dosage forms of the existing immune BCG product.

## Experimental

*Materials*

Live, attenuated strain of *Mycobacterium bovis *(strain 1173P2 of BCG) was developed by Pasteur Institute of Iran and the genetic, phenotypic consistency of Master Seed Lot and Working Seed Lot were evaluated, based on World Health Organization (WHO) recommendations, by the Quality Control Department of Pasteur Institute, Tehran, Iran. Trehalose, glutamate sodium, ferric ammonium citrate, magnesium sulfate 7H2O, potassium hydrogen phosphate (dibasic and monobasic), sodium hydrogen phosphate dibasic, citric acid, ammonium 25%, zinc sulfate 7H2O, methanol and glycerin 87% were provided by Merck Co. (Germany). L-Asparagine monohydrate was purchased from Applichem Co. (Germany), trypton soy agar (TSA), trypton soy broth (TSB), and thioglycolate culture medium were procured from Himedia Co. (India). Lowenstein Jansen’s medium and guinea pigs were gifted by Pasteur Institute of Iran. Rubber stoppers were provided by Helvoet Pharma Co. (Belgium), Aluminium caps and 10R glass vials were purchased from Sarsaz and Pars Ampoul Companies (Iran), respectively. 

*Preparation of samples*

For the growth of BCG, the concentrated Sauton as nutrient culture medium was prepared with L-asparagine monohydrate, zinc sulfate 7H2O, ferric ammonium citrate, magnesium sulfate 7H2O, potassium hydrogen phosphate, citric acid and glycerin 87% in water for injection, sterilized by autoclave (Getinge GE, Sweden) at 121 °C for 20 minutes and finally its pH was adjusted to 7.2 ± 0.1 by ammonium 25%. Incubation (Memert, TV60b, Germany) was performed at 37 ± 0.5 °C for five weeks and the harvested cake was used in the preparation of concentrated bulk after washing with phosphate buffer (pH = 7.38). Bulk solutions were prepared with various amounts of trehalose (2.5%, 5%, and 10% w/v) and transferred into 10R glass vials. In this study, the freeze-dried trehalose-containing formulations were designated F-TH-2.5, F-TH-5 and F-TH-10, whereas the liquid preparations were designated L-TH-2.5, L-TH-5 and L-TH-10. Domestically produced BCG intravesical product, as liquid (L-G) and lyophilized (F-G) forms, was used for the comparative studies. All experiments were performed in triplicate.

*Differential scanning calorimetry (DSC)*

The glass transition temperatures (Tg) of samples were determined using a Differential Scanning Calorimeter (Mettler Toledo Netzsch, 200 F3, Switzerland). Approximately, 10 mg of samples were analyzed in sealed Al-crucibles. The pans were cooled from room temperature to −80 °C at a rate of 10 °C/min, held at −80 °C for 5 min and then heated from −80 °C to 40 °C with a scanning rate of 10 °C/min. 

*Freeze-drying (lyophilization) protocol*

The protocol was designed and developed based on the moisture content, cake appearance and DSC data. The filled vials with half-seated stoppers were loaded into a freeze-drier (Usifroid, SMH 50, France) with a shelf temperature of 5°C. The shelf temperature was reduced to −50 °C at an appropriate rate. The vials were kept frozen at the temperature of −50°C for a predetermined period, followed by heating to −33 °C at a specific rate for primary drying. The temperature was held constant for a few hours, increased to room temperature with the same rate and kept constant again for a few hours. The secondary drying step was carried out at 35 °C at a determined rate and pressure. Finally, the vials were stoppered and sealed by aluminum caps under vacuum, using a vial sealer and stored at 4 °C and room temperature for characterization and stability studies. 

*Determination of bacterial concentration by optical density measurement*

Optical density of all formulations was determined spectrophotometrically (UNICO 2150, USA) at the wavelength of 490 nm, in accordance with WHO and British Pharmacopoeia (BP) (13, 14).

*Safety test (absence of virulent mycobacteria)*

Six healthy guinea pigs, all of the same sex, weighing between 250-400 g with no history of antibiotic intake and negative tuberculin test result were chosen for each set of experiments. Five mg of each formulation was injected subcutaneously. The guinea pigs were carefully monitored for at least 6 weeks. Within this period, if the animals remained healthy, gained weight and showed no signs of progressive tuberculosis, the injected formulation was considered to be free from virulent mycobacterium (13, 14).

*Viability assay*

Each liquid formulation (0.1 mL) or freeze-dried form reconstituted with 3 mL Sauton medium was withdrawn and plated onto Lowenstien-Jansen’s medium. The samples were incubated at 37 ± 0.5 °C for five weeks until colony forming units (CFU) with visible size were developed. The number of viable units per mL was determined by viable counting technique according to BP 2012 (14).

*Assessment of fungal and bacterial contamination*

The accidental fungal and bacterial contaminations of all samples were evaluated by using trypton soy agar (TSA), trypton soy broth (TSB) and thioglycolate culture media incubated at 20-25 °C and 30-35 °C for two weeks, respectively (14).

*Moisture content assay (for lyophilized formulations)*

The residual moisture of the samples was analyzed by coulometric Karl-Fischer titration (Mettler Toledo, DL37 and Switzerland). The method was validated against conventional Karl-Fischer titration. Water from lyophilized samples was extracted by anhydrous methanol. As a reference material, Apura Water Standard 0.01% (Merck) was used and the recovery was considered for the calculation of the residual moisture in the samples. Based on BP requirements, the acceptable moisture content should be less than 3 % in all experiments (15).

*Skin reaction test (for lyophilized formulations)*

Six healthy guinea pigs, each weighing between 250-400 g with no history of any antibiotic intake were selected for this test. Randomly, 0.1 mL of 0.01, 0.1 and 1 mg/mL of reconstituted lyophilized formulations were injected intradermally. The size of the lesions formed at the site of injection after 4 weeks was observed and measured (16).

*Stability studies*

The stability of all freeze-dried and liquid formulations were evaluated at different storage temperatures in accordance with Q5C Guideline of International Conference of Harmonization (ICH Q5C) (17). The accelerated stability tests were executed for 6 months, at room temperature (less than 30 °C) and 4 °C for freeze-dried and liquid preparations, respectively. The stability studies for all samples included survival rate and optical density measurements, safety test, bacterial and fungal contamination test, whereas moisture content determination, skin reaction test and inspection of the cake appearance were only carried out for the freeze-dried formulations. The survival rate for lyophilized formulations was determined by comparing the CFU values (as the main and effective quality parameter) obtained immediately after the freeze drying process with those obtained after 6 months storage at room temperature (25 °C) or 12 months at 4 °C. Similarly, this parameter was determined for liquid formulations through comparison of the viability values at the time of preparation with those obtained after 6 months storage at 4 °C and 12 months at -20 °C. In order to evaluate the effect of lyophilization process on the survival rate, the viability values before and immediately after the freeze drying were also compared for lyophilized samples. 

*Statistical analysis*

Results are reported as mean ± SD. Data obtained were compared using Student’s t-test. Differences between the treatments were assumed to be significant at p < 0.05. Statistical analysis of all data was performed using Statgraphics^®^ centurion, v16.1.11 (2011). 

## Results and Discussion

Commercially available BCG intravesical products which are prepared with different strains of *Mycobacterium bovis *possess various shelf-lives, depending upon the storage conditions. In the United States Pharmacopeia, the stability and shelf-life of this product is reported to be 3 years if stored at 2-8 °C, whereas the British Pharmacopoeia considers a 4-year stability at -20 °C. Intravesical (immune) BCG product which is domestically produced with live, attenuated Pasteur strain 1173P2 of BCG should be stored at less than -10 °C and the mean shelf-life at this condition is labeled 12 months after manufacturing. This product should be thawed and reconstituted before administration. Freeze-thaw process has been found to increase the dead mass and reduce its efficacy. Therefore, as mentioned earlier, the main objective of this investigation was to improve the intravesical BCG formulation in order to ensure that this product could maintain a high proportion of live bacteria, keep stability on milder storage conditions and have reasonable long shelf-life. 

Freeze drying technology could enhance the product stability at room temperature. Although its application is highly recommended for vaccine development, however, microorganism sensitivity to freezing and drying processes is a major problem which may lead to poor survival (18-20). Therefore, lyophilized formulations usually require the inclusion of excipients that stabilize them against damages caused during freezing, drying and upon storage. Among the commonly used pharmaceutical excipients, trehalose has been found application as a stabilizer of microorganisms in undesirable environmental conditions (21-24). Therefore, in the present study, it was decided to replace sodium glutamate which is used as a pharmaceutical excipient in the intravesical BCG formulation with trehalose as an alternative stabilizer and evaluate the effect of this protectant on the stability and efficacy of liquid and freeze-dried BCG products, through the determination of viability value and survival rate.

**Figure 1:**
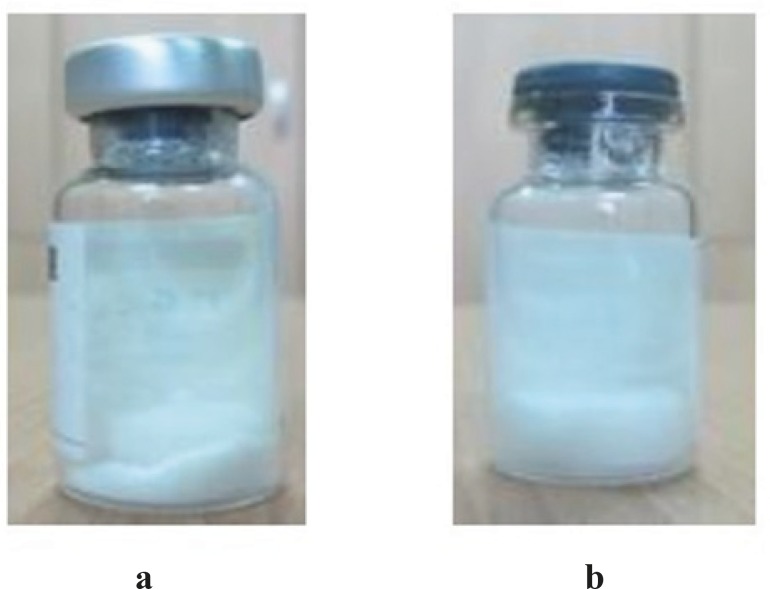
Cake appearance of lyophilized intravesical BCG, a) freeze-dried formulation containing sodium glutamate, b) freeze-dried formulation containing trehalose

Pharmaceutically acceptable lyophilized formulations are required to have desirable characteristics, including rapid and complete reconstitution, acceptable shelf-life and cake appearance as well as full activity following reconstitution. Therefore, it is necessary to determine the temperature below which the desirable properties are guaranteed (25). Differential scanning calorimetry (DSC) is a thermal analysis method which is utilized for the development of lyophilization protocol. In this study, glass transition temperatures (Tg) of samples containing either 1.5% w/v sodium glutamate or 2.5% w/v trehalose were measured and the values, reported as the midpoint of the transition, were found to be -27.4 and -31.8 °C, respectively. These values are of great importance, since the primary drying process should be run below these temperatures to ensure the retention of the structure formed by freezing (25). Figure 1 shows the cake appearance of lyophilized formulations. As depicted, an elegant, consistent cake with no-shrinkage or collapse was observed when the intravesical BCG product was formulated in the presence of trehalose.

Regardless of the type of stabilizer used, the moisture content of all lyophilized formulations prepared in this study was found to be less than 3%, at the time of preparation and after 6 and 12 months storage at room temperature and 2-8 °C which is in consistent with pharmacopoeial requirements and therefore it confirms that the applied freeze-drying conditions were properly chosen. 

Table 1 shows the viability values of freeze-dried BCG formulations containing 1.5% w/v sodium glutamate and 2.5% w/v trehalose before and after lyophilization and the corresponding survival rates following freeze drying and 12 months storage at 4 °C. Table 2 indicates the viability values of liquid BCG formulations containing 1.5% w/v sodium glutamate and 2.5% w/v trehalose at the time of sample preparation and after 12 months storage at -20 °C and the corresponding survival value. As indicated in Table 1, although the survival rates of F-G and F-TH formulations were significantly different immediately after freeze drying (p < 0.05), however, these values for both lyophilized preparations were near to zero following 12 months storage in refrigerator. Regardless of the kind of stabilizer, liquid formulations showed a better stability (p < 0.05) when stored in freezer (-20 °C) for 12 months (Table 2).

**Table 1:** The viability values of freeze-dried BCG intravesical formulations containing 1.5% w/v sodium glutamate and 2.5% w/v trehalose before and after lyophilization and the corresponding survival rates following freeze drying and 12 months storage at 4 °C

	**Viability value before** **lyophilization (× ** **10**6**CFU/mL)**	**Viability value after lyophilization (× 10**6 **CFU/mL)**	**Survival immediately after lyophilization (%)**	**Survival** **after 12 months (%)**	
F-G-01	16.5	0.40	2.42	0
F-G-02	15.8	0.35	2.21	0
F-G-03	12.2	0.10	0.82	0
Mean ± SD (n=3)	14.8 ± 1.8	0.28 ± 0.13	1.82 ± 0.7	-
F-TH-2.5-01	12.9	0.80	6.20	0
F-TH-2.5-02	11.4	0.84	7.36	0
F-TH-2.5-03	10.8	0.51	4.72	0
Mean ± SD (n=3)	11.7 ± 0.88	0.71 ± 0.14	6.09 ± 1.08	-

**Table 2:** The viability values of liquid intravesical BCG formulations containing 1.5% w/v sodium glutamate and 2.5% w/v trehalose at the time of sample preparation and after 12 months storage at -20 °C and the corresponding survival rates

	**Viability value at time zero (× 10**6 **CFU/mL)**	**Viability value after 12 months ** **(× 10**6 **CFU/mL)**	**Survival ** **after 12 months (%)**
L-G-01	16.5	1.42	8.60
L-G-02	15.8	1.54	9.70
L-G-03	12.2	1.07	8.70
Mean ± SD (n=3)	14.8 ± 1.8	1.34 ± 0.2	9.0 ± 0.5
L-TH-2.5-01	12.9	1.47	11.39
L-TH-2.5-02	11.4	1.35	11.84
L-TH-2.5-03	10.8	1.38	12.77
Mean ± SD (n=3)	11.7 ± 0.88	1.4 ± 0.05	12.0 ± 0.57

All formulations were found to be free from bacteria, fungi and virulent mycobacteria indicating no possible interference with stability and survival rate determinations. Results obtained from optical density measurements at time zero and following storage at various temperatures showed values between the accepted criteria (0.47-0.67 and 0.33-0.53 for liquid and freeze dried formulations, respectively). Skin reaction tests were carried out for lyophilized formulations with three different concentrations and the size of the lesions at the site of injection after 4 weeks was observed and measured. The data obtained at time zero and following storage at various temperatures confirmed the safety of the prepared formulations (Table 3).

**Table 3:** Results of the skin reaction test prepared for the lyophilized BCG preparations with three different concentrations, at time zero and following storage at various temperatures.

**time zero**				**after 6 months (room temperature)**			
	1 mg/mL	0.1 mg/mL	0.01 mg/mL	1 mg/mL	0.1 mg/mL		0.01 mg/mL
F2-G	5.8	4.8	3.1	6.8		5.9	4.5
F2-TH-2.5	5.1	4.8	3.1	5.6		4.9	3.2
F2-TH-5	5.2	4.1	3.0	6		5.1	4.2
F2-TH-10	6.9	5.8	4.4	7.5		6.8	4.9
	**time zero**			**after 12 months (refrigerator)**			
	1 mg/mL	0.1 mg/mL	0.01 mg/mL	1 mg/mL	0.1 mg/mL		0.01 mg/mL
F1-G	5.8	4.8	3.1	6.3	5.2		3.7
F1-TH-2.5	5.1	4.8	3.1	5.4	4.9		3.1
F1-TH-5	5.2	4.1	3.0	5.5	4.5		3.4
F1-TH-10	6.9	5.8	4.4	7.1	6		4.7

Accepted criteria: 4.2-7.5, 2.0-6.5 and 0-6.5 mm for 1, 0.1 and 0.01 mg/mL, respectively

This investigation provided some useful information about the role and concentration of stabilizers in the preparation of more efficient and stable intravesical BCG product. Although the trehalose-containing formulations tolerated the harsh conditions of freezing and drying processes much better than those prepared with sodium glutamate, however, both stabilizers failed to maintain the required viability after 12 months storage at 4 °C. Sodium glutamate was capable of stabilizing liquid intravesical BCG, but it also failed to provide an accepted cake integrity once lyophilized. Replacing sodium glutamate with trehalose successfully improved the cake appearance and increased the viability immediately after lyophilization. Furthermore, liquid formulations showed better long term stability during storage at freezing temperature, although the survival rate was significantly higher when trehalose was used. It was also observed that an increase in the content of trehalose resulted in a significant decrease in the viability of both liquid and lyophilized formulations during long term storage. In general, results of this study showed that among the various concentrations applied, 2.5% (w/v) seems to be the appropriate concentration. 

Both sodium glutamate and trehalose are capable of stabilizing microorganisms under harsh environmental conditions. However, trehalose has found more application as a pharmaceutical protectant. Among the advantages reported in the literature for trehalose, one could mention the potential of forming strong hydrogen bonds with water, less hygroscopicity, absence of internal hydrogen bonds (which allows more flexible formation of hydrogen bonds with cells), very low chemical reactivity, the possibility of substitution for water molecules in the membrane during dehydration to maintain its integrity and the capability of protection against heat stress and acting as a “Xeroprotectant” (26-29). Although a better stability against freezing and drying conditions was achieved and higher survival rate was obtained in liquid preparations in the presence of trehalose, however, it is suggested that the effect of a combination of both trehalose and sodium glutamate on the stability and efficacy of intravesical BCG be investigated in further studies. 

## Conclusion

It is very important to ensure that patients suffering from carcinoma in situ (CIS) will receive adequate treatment doses of intravesical (immune) BCG. Therefore, improvement of the storage conditions, viability and survival rates, could guarantee the successful treatment of the disease and reduce the relevant expenses. Our observations supported that using new protectants could be a promising approach to design optimal BCG formulations in terms of storage stability, potency and ease of administration to the patient. However, further studies are required to evaluate various pharmaceutical ingredients or identify stabilizing combinations of excipients in an attempt to increase the number of viable bacillus and in turn the efficacy of the formulation in the treatment of CIS and better prevention of tumor growth.
